# Minimally invasive percutaneous nephrolithotomy guided by ultrasonography to treat upper urinary tract calculi complicated with severe spinal deformity

**DOI:** 10.1590/S1677-5538.IBJU.2015.0408

**Published:** 2016

**Authors:** Zhaohui He, Caixia Zhang, Guohua Zeng

**Affiliations:** 1 Department of Urology, Minimally Invasive Surgery Center, the first affiliated Hospital of Guangzhou Medical University. Guangdong Key laboratory of Urology Guangzhou, China;; 2 Department of Urology, Sun Yat-sen Memorial Hospital, Sun Yat-sen University, China

**Keywords:** Nephrostomy, Percutaneous, Minimally Invasive Surgical, Procedures, Ultrasonography, Urinary Tract

## Abstract

**Objective:**

To report our experience of minimally invasive percutaneous nephrolithotomy(MPCNL) in managing upper urinary tract calculi complicated with severe spinal deformity.

**Materials and Methods:**

Between August 2001 to December 2012, 16 upper urinary calculi in 13 patients with severe spinal deformity were treated by MPCNL. Preoperative investigation of the respiratory function, evaluation of anatomy by intravenous urography (IVU) and CT scan, and preoperative kidney ultrasonagraphy with simulation of the percutaneous puncture were performed in all patients. The percutaneous puncture was guided by ultrasonography.

**Results:**

A total of 19 MPCNL procedures were performed in 16 kidneys, with an average 1.2 procedures in each kidney. Three kidneys needed two sessions of MPCNL, and 2 kidneys needed combined treatment with retrograde flexible ureterscopic lithotripsy. All procedures were successfully completed with no major complications during or after surgery. The mean (range) operative duration was 67 (20-150) min and the mean postoperative haemoglobin drop was 1.0 (0.2-3.1) g/dL. Complete stone-free status was achieved in 14 kidneys. At a mean follow-up of 48(3-86) months, recurrence of small lower calyx stone was detected in one patient. Recurrent UTI was documented by urine culture in two patients and managed with sensitive antibiotics.

**Conclusion:**

PCNL for patients with severe spinal deformities is challenging. Ultrasonography-assisted puncture can allow safe and successfully establishment of PCN tract through a narrow safety margin of puncture and avoid the injury to the adjacent organs. However, the operation should be performed in tertiary centers with significant expertise in managing complex urolithiasis.

## INTRODUCTION

Management of upper urinary tract calculus complicated with severe spinal deformity remains a challenge. For anesthesiologists, the skeletal deformity may compromise pulmonary ventilation, thus complicating anesthesia. Intubation under general anesthesia may also injure the teeth and mandible. Concomitant hypermetabolism may precipitate malignant or non-malignant intra/post-operative fever (1). Bone fragility and the ensuing risk of fractures must also be considered. Moreover, for the urologist, altered urinary anatomy and transposition of visceral organs increase the difficulties of access tract creation and endoscopic manipulation. Due to the potential complications mentioned above and being relatively uncommon in clinics, reports about endoscopic management of upper urinary stones in such patients are rare in the literature (2, 3). Here, we report our experience of using Chinese MPCNL in treating patients with upper urinary stones complicated with severe spinal deformity.

## MATERIALS AND METHODS

From August 2001 to December 2012, 13 patients with upper urinary tract stones with concomitant spinal deformities were treated by MPCNL. 3 patients had bilateral upper urinary calculi, thus the total number of kidneys treated with MPCNL was 16. The therapy modality was approved by the Hospital ethics committee and written informed consent from patients was obtained prior to surgery. The data were collected retrospectively and evaluated (Table-1).

**Table 1:** The patient's characteristics and treatment outcomes.

	1	2	3	4	5	6
Gender/age	F/50	F/42	M/36	F/72	F/75	M/65
Type of scoliosis	Left lumbar	Left thoracolumbar	posterior thoracic	Left lumbar	Right Lumbar	Left lumbar
Renal units	1	1	1	1	1	1
Stone Location	Right pelvis-lower pole partial staghorn	Left proximal ureter	Right pelvis	Left proximal ureter	Left proximal ureter	Left complete staghorn
Stone size (cm)	3.2*1.4	0.6*1.5	2.1*1.3	1.7*0.5	1.3*0.6	6.5*3.5
Respiratory function	mild impaired	Severe impaired	Normal	mild impaired	mild impaired	mild impaired
Op.duration, min	100	20	60	30	45	Two session PCNL, First 90, Second 150
Hb decline, g/dL	1.2	0.2	3.1	0.6	1.2	First1.1; Second 0.8
Stone analysis	No Recorded	No Recorded	No Recorded	No Recorded	No Recorded	Struvite
Follow-up, months	86	84	72	66	48	60
Outcome	NSR, SRF	NSR, SRF	NSR, SRF	NSR, SRF	NSR, SRF, Recurrent UTI	NSR, SRF
	7	8	9	10	11	12

NSR, no stone recurrence; SRF, stable renal function; UTI, urinary tract infection; Hb, haemoglobin; fURS, flexible ureteroscopy; CaOx, calcium oxalate; UA, uric acid; CaPh, calcium phosphate

The study group included 5 males and 8 females. The average patient age was 53 (range 36-76). The types of spinal deformity included 5 lumbar spinal kyphoscoliosis, 2 cases of thoracolumbar spinal kyphoscoliosis, 3 cases of thoracic spinal kyphoscoliosis, 2 cases of posterior lumbar spinal kyphoscoliosis, and 1 case of posterior thoracolumbar spinal kyphoscoliosis complicated with chest deformity. Among them, 3 cases presented also severe hip ankylosis. Cobb angle ranged from 95° to 125°. In eight patients, the affected kidney corresponded to the concave side of the spine, where space for percutaneous access was very limited.

Types of calculi included 4 cases of upper ureteral calculi and 12 renal calculi, among whom 4 had pelvic calculi, 5 had partial staghorn calculi or multiple calculi and 3 complete staghorn calculi. The stone size (maximum diameter on the plain film) ranged from 0.7cm×1.1cm to 5.2cm×7.8cm.

All patients were preoperatively submitted to complete history, clinical examination, routine laboratory blood investigation, coagulation profile, urinalysis and culture, hepatorenal function, and complete evaluation of the respiratory and cardiovascular systems including chest radiography or CT scan, pulmonary function tests, arterial blood gas analysis and echocardiography. Preoperative diagnostic evaluation of the urinary tract consisted of kidneys, ureters, and bladder (KUB) x-ray or intravenous urography (IVU) combined with spiral CT without enhancement and ultrasonography (US). The renal US was performed with simulation of the percutaneous puncture to confirm the feasibility of establishing a percutaneous access.

Respiratory test showed mild restrictive ventilation dysfunction in 8 patients and severe restrictive ventilation dysfunction in 2 patients which were all preoperatively managed with inhalation of compound ipratropium bromide solution. Urine culture demonstrated the colonization of E.coli in three patients and Pseudomonas aeruginosa in one patient and all were managed with sensitive antibiotics.

**Figures 1a and 1b:**
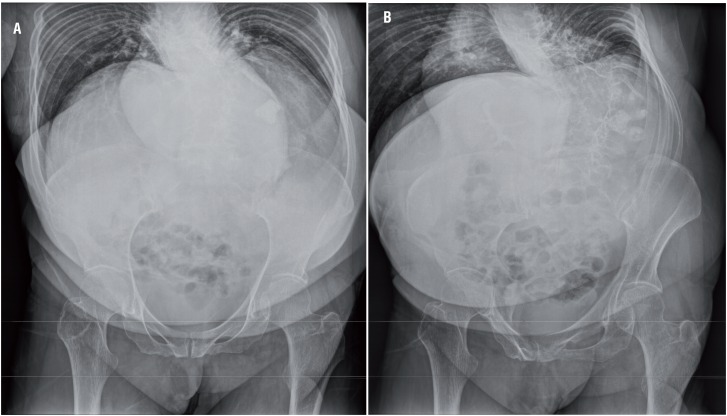
A plain abdominal film (KUB) and IVU show a pelvic and lower calycial stone in left kidney with a good function in a posterior thoracolumbar spinal kyphoscoliosis complicated with chest deformity patient.

**Figures 2a and 2b:**
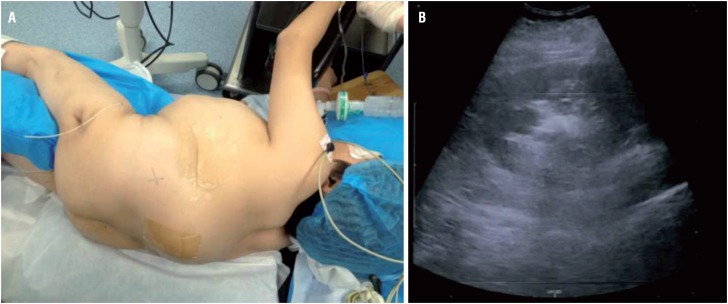
The patient was placed in the lateral decubitus position for the spinal deformity prevented to be placed in the prone position (Figure-2a). A successful US-guided puncture was performed. The dotted line corresponds to the tract of needle (Figure-2b).

**Figure 3:**
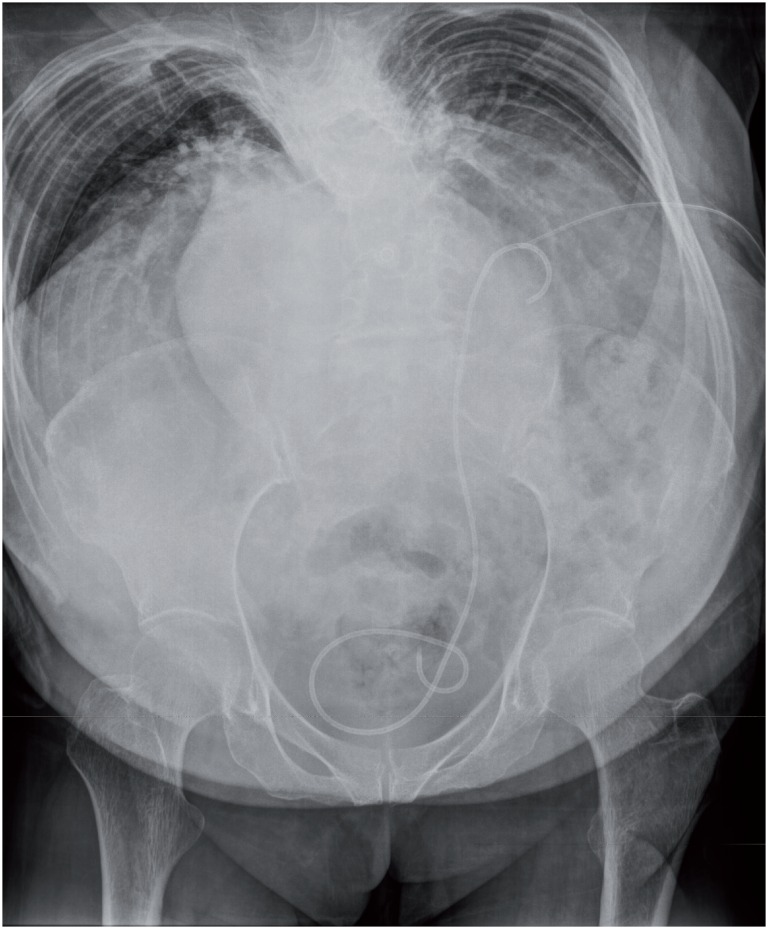
KUB after MPCNL: the stone was removed completely and a ureter stent and a nephrostomy tube were placed in the left kidney.

The surgical technique of the Chinese MPCNL has been described in previously published articles (4–6). Following general anesthesia and intubation, with the patient in a lithotomy position, a 5F open tip ureteral catheter was placed in the ipsilateral ureter under cystoscopy. In 9 cases, the patient was then positioned in prone position with adequate protection to avoid compression of any spinal or bony protrusions. Two patient had to be repositioned in the lateral decubitus position (Figures 1-3) and two patients were placed in the supine position because decreased blood oxygen saturation in the prone position or their spinal deformity prevented them to be placed in the prone position. A US-guided fluoroscopic confirmed puncture was performed to the most appropriate calyx based on preoperative imaging and patient positioning. After the puncture was confirmed successful, a 0.035-inch guidewire was inserted into the collecting system. The percutaneous tract was then dilated to 18F or 20F with fascial dilators (Cook Urological, Spencer, IN), and a same sized peel-away sheath was placed as the percutaneous access port. Subsequently, a 8/9.8F semirigid ureteroscope (Richard Wolf, Knittlingen, Germany) or a 8.5/12.5F nephroscope (Lixun Nephroscope, Richard Wolf, Knittlingen, Germany) was used for nephroscopy. The stone was fragmented by pneumatic lithotripsy or holmium: YAG laser. The larger fragments (0.3cm-0.5cm) were extracted with a 5F forceps (Richard Wolf, Knittlingen, Germany), and the fragments<0.3cm were mainly flushed out with an endoscopic pulsed perfusion pump. In cases with complex calculi, like staghorn calculi or stones located in diffused multiple calices, where multiple tracts was thought to be necessary, the multiple tracts (being two tracts in most of the cases) were created in the same session based on the stone configuration and collecting system anatomy.

Finally, a 5F or 6F Double-J stent was inserted in the ureter, and a same caliber nephrostomy tube (18F-20F) was left in the collecting system. If the patient presented with febrile urinary infection or purulent pelvic urine was aspirated during puncture, a nephrostomy tube was placed initially, and the MPCNL was performed after antibiotic treatment and drainage for 1 week.

KUB radiography, nephrostography or CT scan were performed 24 to 48 hours after surgery to assess the stone free status. In cases with significant residual stones, a second-staged MPCNL for stone size >2cm or flexible ureteroscopic lithotripsy for stone size <2cm was performed 5 to 7 days later. The second stage MPCNL was performed through a new percutaneous access tract if required. If a second-stage surgery was unnecessary, the nephrostomy tube would be removed on postoperative day 3 to 5 when the drainage was clear, and the Double-J stent would be extracted 2 to 3 weeks postoperatively. The operative time was calculated from puncture to placement of the nephrostomy tube. The change in hemoglobin concentration was estimated by comparing preoperative and 48 hours postoperative routine blood test.

## RESULTS

A total of 19MPCNLs were performed in 16 kidneys of 13 patients affected by spinal deformity, with an average 1.2 procedures in each kidney. Three kidneys needed two sessions of MPCNL, and 2 kidneys needed combined treatment with retrograde flexible ureterscopic lithotripsy. Multiple tracts were performed in 6 kidneys, with the average 1.38 tracts per kidney. In two patients a nephrostomy tube was placed for 1 week before the MPCNL as they had purulent pelvic urine. The average operative time of each MPCNL procedure was 67 (range 20-150) minutes. The mean hemoglobin drop was 1.0 (0.2-3.1) mg/dL and no blood transfusions were required. Radiography was performed on the second postoperative day. Complete stone-free status was achieved in 14 kidneys. No complication was noted during or after surgery. Stone analysis was performed in 8 patients and showed calcium oxalate in 3 patients, calcium oxalate with uric acid and calcium phosphate in two, and struvite in three.

The mean follow-up was 48 (3-86) months. A recurrent stone was detected in lower calyx in one patient but since it was asymptomatic no specific management was done. The serum creatinine level was stable at 0.9-1.2mg/dL in all patients (normal 1.3mg/dL at our institutions). Recurrent UTI with the colonization of E. Coli was documented by urine culture in two patients and were managed with sensitive antibiotics.

## DISCUSSION

The spinal column has four physiological curves in the sagittal plane, whereas no curve should be observed on the coronal plane. The curve to either side refers to spine scoliosis and the severe scoliosis is generally defined as a Cobb angle above 90° (6). Severe scoliosis may be associated with distortion of the chest cavity, pelvis and compression of peritoneal organs, thus altering the anatomical location of internal organs. In case of severe deformity the altered conformation of the rib cage and restricted lung ventilation often cause respiratory dysfunction. In this study group, 10 patients (77%) were found to have mild or severe restrictive ventilation dysfunction, so for these patients the preoperative pulmonary function tests and arterial blood gas analysis were performed.

The effect of altered anatomy to the urinary system may lie in possibly urinary obstruction, further promoting formation of urinary calculi. Theoretically the risk of urinary stone disease is higher in these patients, with reported incidence rates of up to 20%. However, Vetter U et al. reported an incidence of 4.7-6.9% (6/127 and 4/58, respectively) in children with osteogenesis imperfecta, which did not appear to differ from that seen in the general population (7, 8).

As is the case with the general population, small renal calculi complicated by spinal deformity can be treated using the flexible ureteroscope, while larger stone-burden still required intervention of PCNL or an open procedure (3). The challenge of PCNL in such patients, in the presence of aberrant anatomy, lies in the establishment of an appropriate percutaneous pathway and avoidance of adjacent organ injury. The establishment of PCN pathway with fluoroscopic guidance alone in such patients is relatively risky or less feasible, requiring the assistance of ultrasound or laparoscopy. The ultrasound guidance allows the safe establishment of a PCN tract in a narrow safety margin of puncture due to the anatomic alteration or abnormal anatomic structure and avoidance of injuring the neighboring organs. The experience of other authors has supported the superiority of US over fluoroscopy in guiding PCNL in special patients. Desai et al. successfully treated nine patients with ectopic renal calculi by using US assisted PCNL puncture (9).

In the case of less experience in ultrasonography-assisted percutaneous renal puncture or evident space-occupying organs present around the pre-established PCN pathway, laparoscopy-assisted PCNL can be used. The laparoscopic assistance allows the intentional avoidance or separation of surrounding organs, further preventing the injuries of neighboring organs. In 1985, Eshghi et al. first described the technique of laparoscopy assisted PCNL for ectopic pelvic kidneys (10). Since then, several authors have reported successful experiences with the technique (11, 12). Recently, using the laparoscopic method, Seref B et al. safely removed a 11.9mm stone from the pelvis of the right kidney in a patient with osteogenesis imperfecta (13). For cases in which there is absence of obvious space-occupying organs around the pre-establishment of PCN access, we prefer ultrasonography-assisted puncture. In our extensive experience with the Chinese MPCNL however, we prefer US assisted puncture. Not only does this allow visualization of adjacent organs, thus minimizing inadvertent injuries, but it also allows accurate puncture through a calyceal fornix, thereby reducing the intraoperative bleeding. Although the laparoscopy-assisted PCNL can be easily performed, it can't ensure an accurate percutaneous calyceal pathway. In this group all 19 MPCNLs were performed with this procedure and the mean hemoglobin drop for each MPCNL procedure was only 1.6mg/dL and no surrounding organ injuries happened, suggesting the safety of such procedure.

For the complete staghorn stones or complex calculi which obviously had the possibility of requiring multiple tracts, we preferred to establish two or three tracts at the beginning of the surgery based on the configuration of stone and collecting system. In most of the cases the secondary tract was established meanwhile, but through the third puncture we always only put the guidewire in the collecting system without dilating initially. We only dilated the third puncture if the surgery was smooth and endoscopic manipulation time was no more than 90 minutes. We preferred that establishment of multiple tracts at the ontset of the surgery because there is no extravasation or bleeding allowing easy and accurate ultrasound-guided puncture. Obviously, the potentially intraoperative leak or hemorrhage would increase the difficulty in puncturing under the ultrasound and decrease the accuracy in establishing a new percutaneous tract. Furthermore, the simultaneous use of multiple tracts can accelerate the removal of stone fragment and shorten the operating time, in addition to reducing the risk of urosepsis by lowering the renal pelvic pressure (4, 14). Finally, it must be admitted that this paper is limited to a retrospective study; the collection of data prospectively may evaluate this technique more objectively.

## CONCLUSIONS

Complex upper urinary tract calculus complicated with spinal deformity represents a challenge to anesthesiologists and urologists. These patients always need PCNL intervention, the difficulty of PCNL in such patients lies in establishing appropriate PCN pathway and preventing injuries of the adjacent organs. When there is no presence of obvious space-occupying organs around the pre-establishment of PCN access, ultrasonography-assisted puncture can allow safely and successfully establishment of PCN pathway in a narrow safety margin of puncture due to the anatomic alteration, but the operation should be performed in tertiary centers with significant expertise in managing complex urolithiasis.
